# Effects of Spaceflight on Bone Microarchitecture in the Axial and Appendicular Skeleton in Growing Ovariectomized Rats

**DOI:** 10.1038/srep18671

**Published:** 2015-12-22

**Authors:** Jessica A. Keune, Adam J. Branscum, Urszula T. Iwaniec, Russell T. Turner

**Affiliations:** 1 Skeletal Biology Laboratory, School of Biological and Population Health Sciences, Oregon State University, Corvallis, OR 97331, USA; 2 Biostatistics Program, School of Biological and Population Health Sciences, Oregon State University, Corvallis, OR 97331, USA; 3 Center for Healthy Aging Research, Oregon State University, Corvallis, OR 97331, USA

## Abstract

This study investigated the effects of a 14-day spaceflight on bone mass, density and microarchitecture in weight bearing (femur and humerus) and non-weight bearing (2^nd^ lumbar vertebra and calvarium) bones in the context of ovarian hormone insufficiency. 12-week-old Fisher 344 rats were ovariectomized 2 weeks before flight and randomized into one of three groups: 1) baseline (n = 6), 2) ground control (n = 12) or 3) spaceflight (n = 12). Additional ground-based ovary-intact rats provided age-matched reference values at baseline (n = 8) and landing (n = 10). Ovariectomy resulted in bone- and bone compartment-specific deficits in cancellous bone volume fraction. Spaceflight resulted in lower cortical bone accrual in the femur but had no effect on cortical bone in the humerus or calvarium. Cancellous bone volume fraction was lower in flight animals compared to ground control animals in lumbar vertebra and distal femur metaphysis and epiphysis; significant differences were not detected in the distal humerus. Bone loss (compared to baseline controls) in the femur metaphysis was associated with lower trabecular number, whereas trabecular thickness and number were lower in the epiphysis. In summary, the effect of spaceflight on bone microarchitecture in ovariectomized rats was bone-and bone compartment-specific but not strictly related to weight bearing.

The form and function of biological systems, including the skeleton, have evolved on Earth in the presence of a nearly uniform gravitational field; the skeletons of terrestrial vertebrates are subjected to large but transient (dynamic) mechanical loads that oppose the gravitational vector during normal activities such as walking^1^,^2^. Because of its fundamental importance to skeletal function, absence of gravitational-mediated dynamic loading during spaceflight results in altered bone mass and microarchitecture. However, because the loading pattern normally experienced by the skeleton is bone- and bone compartment-specific, a uniform skeletal response to a change in gravity is not anticipated. This concept is supported by the observation that astronauts typically lose bone during long-duration orbital spaceflight, but the pattern of bone loss shows considerable individual as well as site-specific variation^3^,^4^,^5^. Similar large variation in the magnitude of bone loss was noted in young healthy males following 3 months of bed rest^6^, an earth-based model for reduced dynamic skeletal loading.

As in humans, the impact of spaceflight on the skeleton of animals has been highly variable^5^. Also, the negative effects of spaceflight on the skeleton are not due solely to decreased dynamic mechanical loading. Centrifugation (1G) in orbit, for example, was ineffective in preventing development of osteopenia in rats^7^ and spaceflight actually resulted in an increase in bone size in calvaria of growing mice^8^. Inability to precisely predict the magnitude and location of bone loss in astronauts or laboratory animals suggests that multiple factors influence the skeletal response to spaceflight.

Gonadal hormone levels vary among individuals and change through the menstrual cycle in women. Reproductive changes associated with spaceflight have not been systematically studied in women but in mice spaceflight induced cessation of cycling, loss of corpora lutea, and reduced estrogen receptor mRNA levels in the uterus^9^. Similarly, hindlimb unloading disrupted cycling in rats^10^. Estrogens represent an important class of factors known to influence the skeletal response to dynamic mechanical loading^11^,^12^. These hormones are important regulators of bone growth and turnover^13^ and are often reduced in rats following spaceflight or simulated spaceflight^14^,^15^,^16^. Ovarian hormone insufficiency has well-characterized skeletal effects in growing female rats^17^,^18^,^19^. Specifically, ovariectomy (ovx) results in cancellous osteopenia and increased radial and longitudinal bone growth. Furthermore, the skeletal abnormalities are replicated following administration of the potent antiestrogen ICI 182,780^20^ and are prevented in ovx rats by replacement with natural as well as with synthetic estrogens, including ones that cannot be metabolized to other classes of steroid hormones (e.g., diethylstilbestrol). Importantly, administration of estrogens attenuates the detrimental changes in a variety of animal models for skeletal disuse^21^,^22^,^23^. Taken together, these findings suggest that there is a positive interaction whereby estrogen enhances the skeletal response to dynamic loading. Ovx results in a general increase in the rate of bone turnover, but bone loss occurs primarily at skeletal sites experiencing low strain energy^11^. As a consequence, it would be anticipated that skeletal unloading during spaceflight would amplify ovx-induced bone loss.

To our knowledge, STS-62 is the only spaceflight mission where bone changes in gonadal hormone-deficient animals have been assessed^24^. We evaluated the effects of this 14-day spaceflight on bone growth, gene expression and bone turnover in sexually mature but slowly growing ovx rats. Spaceflight reduced bone accrual in tibial diaphysis by inhibition of periosteal bone formation. The suppressive effects of spaceflight on cortical bone formation in the tibial diaphysis contrasted with a lack of an effect on cancellous bone formation in the proximal tibial metaphysis. However, there was net cancellous bone loss due to increased bone resorption. Because evaluation of bone microarchitecture in this study was limited to the tibia, it is not clear whether the observed effects are generalizable to other skeletal sites. In this follow-up study, we took advantage of archived specimens obtained from the STS-62 mission to evaluate the combined effects of the spaceflight and ovx-induced gonadal hormone depletion on bone mass, density and microarchitecture in representative bones from the appendicular weight bearing (femur and humerus) and axial non-weight bearing (lumbar vertebra and calvarium) skeleton.

## Results

### Effects of Spaceflight on Bone Mass and Microarchitecture of Femur

**Figure 1:**
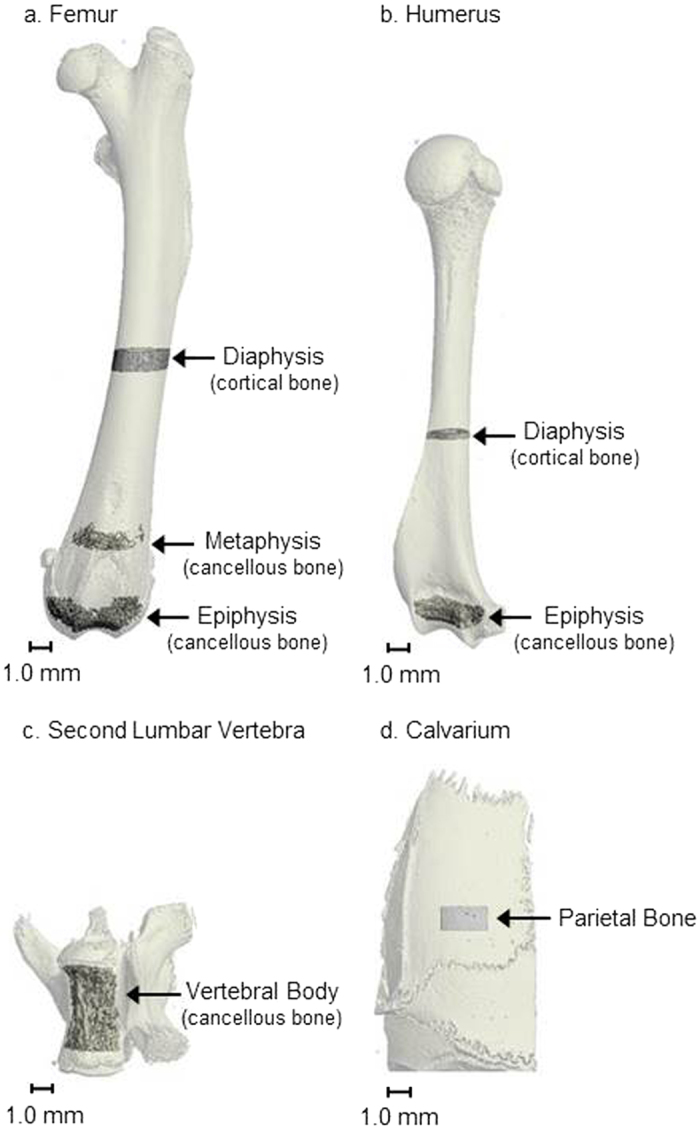
Regions of interest analyzed in (a) femur, (b) humerus, (c) second lumbar vertebra and (d) calvarium.

The effects of spaceflight on femur length, density and microarchitecture (Fig. 1a) are shown in Table 1. Reference values for asynchronous, age-matched, ovary-intact rats are also provided in the table.

#### Ovary-intact reference

Femora were longer and femur area, BMC and BMD were greater in 14-week-old compared to 12-week-old ovary-intact rats. Analysis of cortical bone in the femur diaphysis showed cortical area and cortical thickness to be greater in the older animals. However, significant differences in femur cross-sectional area, marrow area or polar moment of inertia were not detected between the two age groups. Analysis of cancellous bone in the distal femur metaphysis showed greater structure model index and trabecular thickness and a tendency (p = 0.07) for greater cancellous bone volume fraction in the 14-week-old compared to the 12-week-old animals. However, significant differences in connectivity density, trabecular number and trabecular spacing were not detected between the two age groups. Analysis of cancellous bone in the distal femur epiphysis showed a tendency (p = 0.07) for lower structure model index in the older compared to the younger age group. Significant differences between the two groups were not detected in any of the other epiphyseal endpoints evaluated.

#### Spaceflight

**Figure 2:**
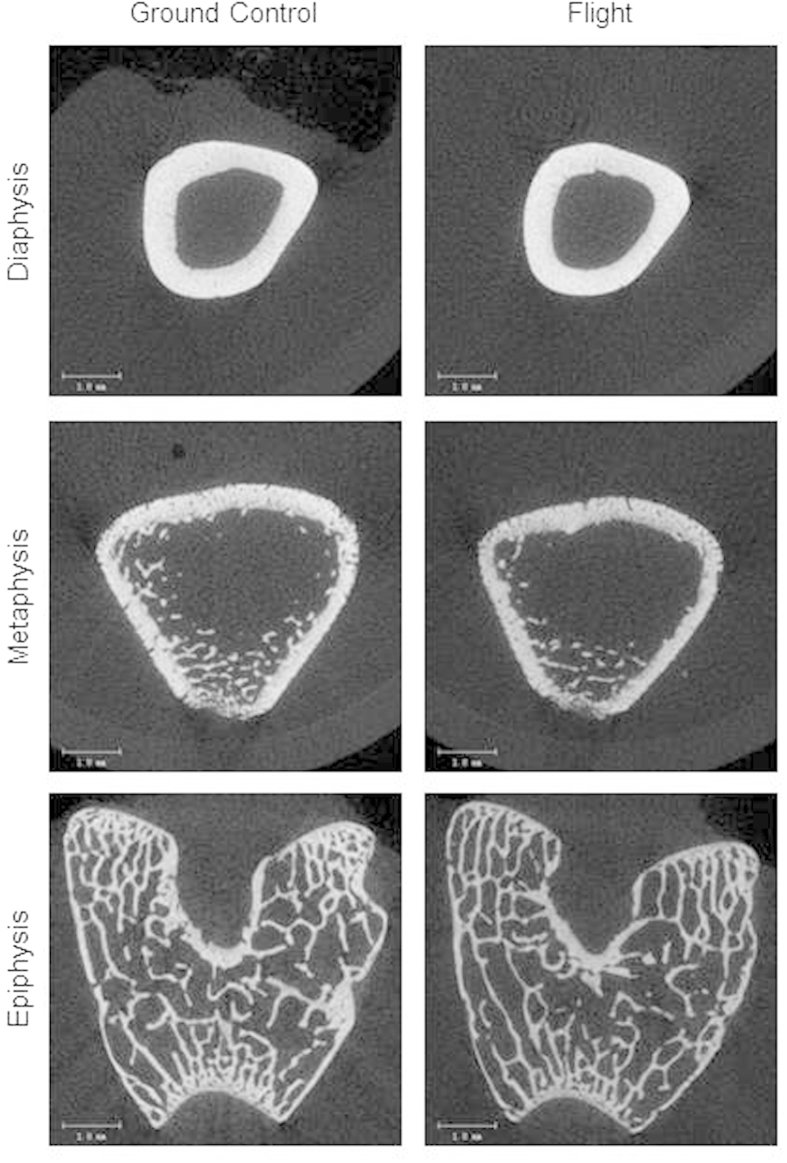
Representative microcomputed tomography images from femur. Diaphyseal microarchitecture did not differ but, compared to ground controls, flight animals had lower cancellous bone volume fractions in metaphysis and epiphysis. Scale bar is 1 mm.

Femora were longer in ground control and flight rats compared to baseline rats. Significant differences in femur length were not detected between the flight and ground control animals. Flight rats had lower BMC compared to the ground controls and lower BMD compared to both baseline and ground control rats. In the femur diaphysis, cortical thickness in the ground controls was greater compared to the baseline group. Significant differences between the three groups were not detected for any of the other cortical endpoints assessed. In the femur metaphysis, flight rats had lower cancellous bone volume fraction and connectivity density compared to the baseline and ground control groups. The structure model index was greater in the flight group compared to both baseline and ground controls. Additionally, the flight group had a lower trabecular number and greater trabecular spacing compared to the baseline group. In the femur epiphysis, the flight group had lower cancellous bone volume fraction, connectivity density, trabecular number and trabecular thickness compared to baseline and ground control rats, and had greater structure model index and trabecular spacing. Compared to baseline controls, the ground control rats also had lower cancellous bone volume fraction and connectivity density, while the structure model index and trabecular spacing were greater. Images representing the observed effects of spaceflight on cortical and cancellous bone in the femur are shown in Fig. 2.

### Effects of Spaceflight on Bone Mass and Microarchitecture of Humerus

The effects of spaceflight on humerus length, density and microarchitecture (Fig. 1b) are shown in Table 2. Reference values for asynchronous, age-matched, ovary-intact rats are also provided in the table.

#### Ovary intact reference

Humeri were longer in 14-week-old compared to 12-week-old rats. A tendency (p = 0.07) for greater bone area and BMC was observed in the 14-week-old compared to the 12-week-old animals. Significant differences were not detected in BMD. Analysis of the cortical bone in the humerus diaphysis showed lower marrow area in the older animals. However, significant differences in humerus cross-sectional area, cortical area or polar moment of inertia were not detected between the two age groups, and only a trend (p = 0.07) for greater cortical thickness was observed in the older rats. Analysis of cancellous bone in the humerus epiphysis showed no significant differences between age groups in any of the endpoints evaluated.

#### Spaceflight

**Figure 3:**
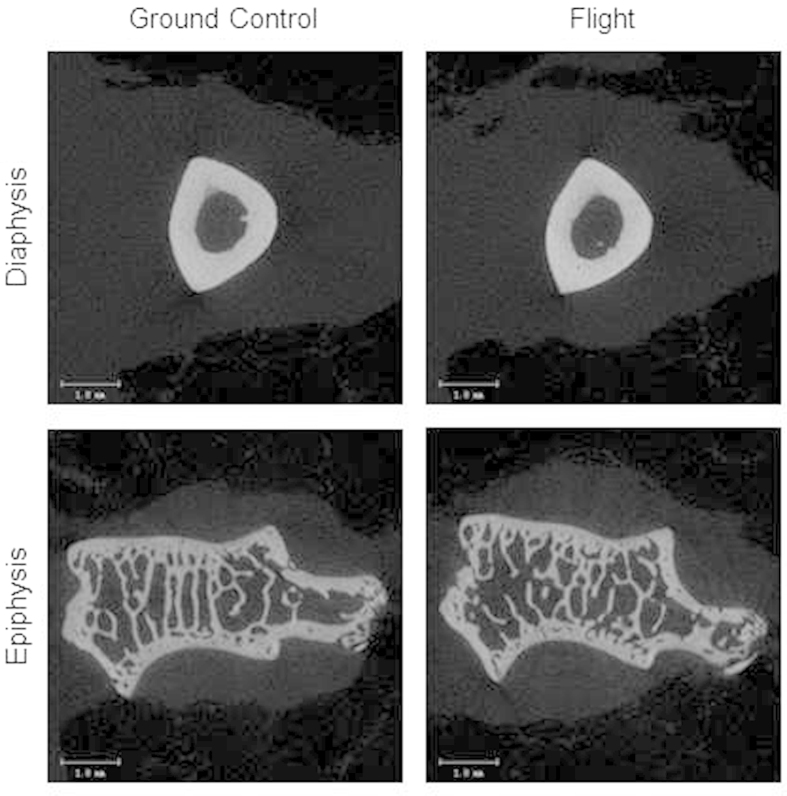
Representative microcomputed tomography images from humerus. Cortical and cancellous microarchitecture did not differ between ground control and flight animals. Scale bar is 1 mm.

Spaceflight had no significant effect on humerus length or bone area, BMC and BMD. In the humerus diaphysis, spaceflight resulted in greater marrow area. However, significant differences in cross-sectional area, cortical area, cortical thickness or polar moment of inertia were not detected with flight. In the humerus epiphysis, significant differences between the ground controls and flight animals were not detected for any cancellous endpoint evaluated. Images representing the lack of an effect of spaceflight on cortical and cancellous bone in the humerus are shown in Fig. 3.

### Effects of Spaceflight on Bone Microarchitecture of Lumbar Vertebra

The effects of spaceflight on cancellous bone microarchitecture (Fig. 1c) in the second lumbar vertebra are shown in Table 3. Reference values for asynchronous, age-matched, ovary-intact rats are also provided in the table.

#### Ovary intact reference

Connectivity density and structure model index were lower in the older animals, but no significant differences in cancellous bone volume fraction, trabecular number, trabecular thickness or trabecular spacing were detected between the two age groups.

#### Spaceflight

**Figure 4:**
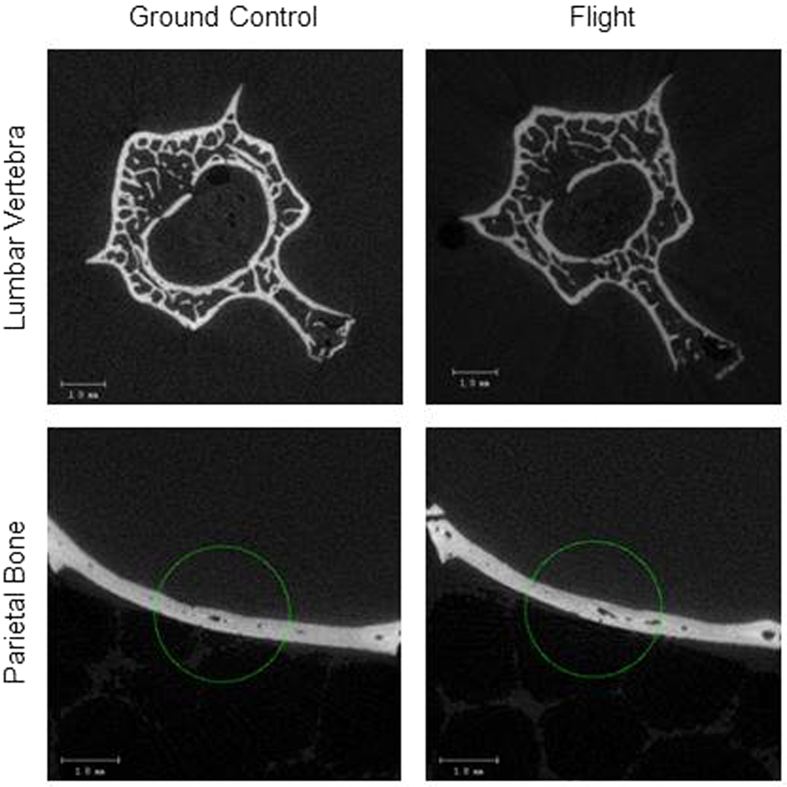
Representative microcomputed tomography images from lumbar vertebra and parietal bone. Compared to ground controls, flight animals had lower cancellous bone volume fraction in lumbar vertebra. However, differences in calvarial thickness were not detected between the groups. Scale bar is 1 mm.

Flight rats had lower cancellous bone volume fraction, trabecular number and trabecular thickness compared to ground controls, and had a greater structure model index and trabecular spacing. Connectivity density did not differ between flight and ground control rats. Images representing the effects of spaceflight on cancellous bone in the lumbar vertebra are shown in Fig. 4.

### Effects of Spaceflight on Thickness of Parietal Bone of Calvarium

The effect of spaceflight on parietal thickness (Fig. 1d) is shown in Table 3. Spaceflight had no significant effect on thickness of the parietal bone. Images representing the lack of effect of flight on parietal thickness are shown in Fig. 4.

## Discussion

Recent technological advances in routine nondestructive imaging of bone (high resolution μCT for evaluation of 3-dimensional bone microarchitecture) correspond with a decline in spaceflight opportunities. The present analysis, addressing the skeletal consequences of a 14-day spaceflight in sexually mature slowly growing ovx rats with established osteopenia, represents the first large scale μCT-based study of the effects of spaceflight on 3-dimensional bone microarchitecture. The study takes advantage of the relatively large number of ovx animals flown on STS-62 and the variety of archived bones representative of the axial and appendicular skeleton normally subjected to differing levels of dynamic weight bearing. It provides a more complete picture of bone-specific changes in bone mass, density and microarchitecture in gonadal hormone-deficient animals following spaceflight.

The time course for cancellous bone loss in the proximal tibia metaphysis of ovx Fisher 344 rats in the present study was previously evaluated by histomorphometry^24^. Rapid bone loss occurred during the initial two weeks following surgery. However, cancellous bone area fraction subsequently stabilized and further bone loss was minimal. The present μCT analysis detected a similar response in the distal femur metaphysis. However, a different pattern was observed in the distal femur epiphysis where further bone loss associated with ovx was apparent in the ground controls during the flight interval. Spaceflight resulted in additional bone- and bone compartment-specific changes. Compared to baseline, spaceflight reduced femur BMD, slowed or prevented an age-related increase in bone area, BMC and cortical thickness, but had no effect on the increase in femur length. Cancellous bone volume fraction in the distal femur metaphysis and epiphysis were decreased compared to baseline or ground controls. Compared to ground controls, spaceflight had no effect on cortical and cancellous bone measurements in the humerus, or on calvaria thickness. In contrast, flight animals had lower cancellous bone volume fraction in vertebral body.

There is precedent for independent actions of estrogen^25^ and weight bearing^26^ on bone mass. Ovx combined with immobilization resulted in a further decrease in bone mineral density compared to either ovx or immobilization alone^27^. On the other hand, treadmill exercise or direct loading of rat hindlimbs^12^ reduced cancellous bone loss in ovx rats compared to that in sedentary controls^11^. The results in disuse models are consistent with our spaceflight findings regarding changes in bone mass.

Bone and bone compartment differences in the skeletal response to spaceflight have been reported previously^5^. Differences in gender, age of the animals flown, duration of spaceflight, number of animals studied, strain of rat, housing conditions and region of interest measured have contributed to the variability^5^. However, the scope of the present study provides strong evidence that spaceflight results in bone- and bone compartment-specific effects on bone growth and turnover independent of most of the common variables.

Total bone mass consists of cortical and cancellous compartments, where cortical bone makes up the majority of bone and primarily serves a structural role, whereas cancellous bone serves a dual function, structure and maintenance of mineral homeostasis. Most spaceflight studies have been performed using growing male rats^5^. Radial (periosteal) bone growth is often reduced in young male rats flown in space^28^,^29^,^30^,^31^,^32^,^33^ resulting in decreased accumulation of cortical bone. However, as shown for the femur in the present study in females, no reduction was noted compared to prelaunch baseline values. The effects of spaceflight on cancellous bone have been highly variable, with either no change or decreased cancellous bone volume fraction reported^34^,^35^. The present study suggests that the observed variability documented in the literature is due in part to differences in the response of individual cancellous compartments to spaceflight.

Tibiae and femora are subjected to high levels of dynamic skeletal loading during normal weight bearing activities. Ovx is associated with increased bone turnover in the proximal tibial epiphysis, but this compartment is normally resistant to bone loss, presumably because of high prevailing mechanical strain energy levels during normal weight bearing^11^. In the present analysis, we detected a cancellous bone volume fraction reduction and microarchitectural changes in the distal femur epiphysis associated with ovx and exaggerated by spaceflight. Specifically, spaceflight resulted in changes in bone microarchitecture consistent with deterioration in bone quality (lower connectivity density, trabecular number and trabecular thickness and higher structure model index and trabecular spacing). The bone loss is notable because the epiphysis is crucial to normal skeletal mechanical function.

In addition to femur, we evaluated humerus, lumbar vertebra and calvarium. The reported effect of spaceflight on the humerus of growing male rats appears to depend on flight duration. Reduced bone formation was detected following 10- and 14-day flights but not following a 4-day flight, and lower cancellous bone area fraction was detected after the 14-day flight but not after 4- and 10-day flights^34^,^35^. Some, but not all, studies suggest that longitudinal and radial bone growth is impaired in the humerus of growing male rats during spaceflight^36^,^37^,^38^,^39^. In the present analysis, most indices of total, cortical and cancellous bone mass and microarchitecture in the humerus of ovx rats were impacted by ovx but not by spaceflight. Although vertebrae are not considered to be weight bearing in the rat, they are load bearing. Changes in cancellous bone volume fraction or fluorochrome labeling were not detected in vertebra of growing male rats following 7- and 14-day spaceflights^33^,^40^. Other studies suggest that spaceflight results in vertebral disc abnormalities^41^. The present analysis detected the expected effects of ovx on vertebral cancellous bone volume fraction^42^ and spaceflight resulted in further deterioration in most measured endpoints. Calvaria form by intramembranous ossification and are not weight bearing bones. However, the upward fluid shift during spaceflight may result in an increase in mineralization^39^. Histological and biochemical abnormalities were reported for the skull following some but not all spaceflights^39^,^43^,^44^. Additionally, a decrease in mRNA levels for bone matrix proteins occurred in rat calvaria^28^. Simmons *et al.*^45^ reported slight decreases in calcium and magnesium concentrations in calvaria following spaceflight, which may have reflected decreased bone formation. The present μCT analysis failed to detect an effect of spaceflight on calvarium thickness. Thus, in the context of ovarian hormone deficiency, the humerus and calvaria appear to be more resistant to the detrimental skeletal effects of spaceflight than long bones in the hindlimb (tibia and femur) or vertebra, indicating that the skeletal response to spaceflight is not strictly related to magnitude of weight bearing.

The present analysis focused on ovx- and spaceflight-induced changes in bone mass, density and microarchitecture in representative weight bearing and non-weight bearing bones. However, detailed histomorphometric evaluation of the tibial diaphysis and proximal metaphysis has been performed^24^. Additionally, mRNA was isolated from periosteum (pooled tibia and femur) and proximal metaphysis (tibia) and analyzed for expression of bone matrix proteins, growth factors and cytokines^24^,^46^. Based on static and dynamic bone histomorphometry and gene expression analysis we concluded that spaceflight slowed periosteal but not cancellous bone formation and increased cancellous bone resorption. These findings provide cellular mechanisms for the reduced accrual of total and cortical bone and cancellous bone loss in weight bearing bones of the hindlimb of ovx rats during spaceflight. Normal female rats have not been flown in space. The skeletal effects of near weightlessness on female rats with normal gonadal function are, therefore, unknown. However, ground-based studies using the hindlimb unloading model reported that skeletal unloading has similar effects on bone formation and bone resorption in 6-month-old male and female rats with intact gonadal function^47^. Hindlimb unloading was developed as a ground based model for spaceflight to circumvent the limited number of spaceflight opportunities^48^. The strengths of this model are its ease of application, replication of fluid shifts characteristic of spaceflight and functional unloading of the hindlimbs. The skeletal changes in tibia observed in ovx flight rats were later replicated using this model^49^. Unfortunately, hindlimb unloading is not useful for modeling the effects of spaceflight on forelimbs or vertebrae because these remain dynamically loaded.

Although there have been no recent experiments evaluating the effects of spaceflight on bone microarchitecture in rats there have been studies evaluating mice. A 3-month spaceflight study was performed on the international space station using growing male wild type and pleiotrophin-overexpressing mice. Unfortunately, only 1 wild type and 2 mice overexpressing the transgene survived the flight^50^. Thus, the small number of surviving animals limits generalization of the results and raises concern regarding contributing effects of housing conditions. A 15-day spaceflight (STS-131) resulted in decreased cancellous bone volume fraction, increased osteocyte canalicular volume, and evidence for cell cycle arrest during osteogenesis in adult female mice^51^. Spaceflight also resulted in an increase in bone volume in calvaria in these mice^8^. However, in contrast to the present study, spaceflight was associated with weight loss. This may be important because weight loss results in decreased bone formation, increased bone resorption and cancellous bone loss in adult rodents^52^. Thus, nutritional deficiency as well as microgravity may have contributed to the skeletal effects of spaceflight in the STS-131 mouse studies.

In summary, in the context of established ovarian hormone deficiency, a 14-day spaceflight resulted in bone- and bone compartment-specific decrements in bone acquisition and a negative turnover balance leading to deficits in bone mass and defective microarchitecture. The observed changes illustrate the importance of evaluating multiple bones and bone compartments as well as the limitations of ground-based models for spaceflight.

## Methods

### Experimental Design

Twelve-week-old female Fischer 344 rats (Taconic Farms, Germantown, NY) were used in the study. The animals were maintained in accordance with the NIH Guide for the Care and Use of Laboratory Animals and the experimental protocol was approved by the NASA and Mayo Clinic Animal Care and Use Committees. The rats were ovx 14 days prior to launch and randomly assigned to one of three treatment groups: 1) baseline control (baseline, n = 6), 2) ground-based flight control (ground control, n = 12), or 3) spaceflight (flight, n = 12). The baseline group was sacrificed on day of launch. The flight animals were flown on the Space Shuttle Columbia (STS-62) for 14 days. Flight and ground control animals were housed in animal enclosure modules (AEM) with 6 rats/AEM. The AEMs were maintained at 28 °C. All animals were provided with food (Teklad L356 food bars; Teklad Inc., Madison, WI) and water *ad libitum*. Flight animals were sacrificed 4-6 hours after landing. Additional ground-based ovary-intact rats (n = 18) were evaluated. The ovary-intact animals represent reserve rats to be used if the originally planned flight had been delayed. Since the mission was carried out as anticipated, these animals were maintained in a vivarium and sacrificed to provide age-matched reference values for baseline (n = 8, 12 weeks old) and flight (n = 10, 14 weeks old). The purpose of the ovary-intact controls was 2-fold: 1) to evaluate normal age-related changes in bone over the 2 week flight period, and 2) to verify that ovx resulted in anticipated cancellous osteopenia. All rats were sacrificed by decapitation and bones were harvested and placed in 70% ethanol for analysis.

### Densitometry

Total femur and humerus bone mineral content (BMC, mg), bone area (mm^2^) and bone mineral density (BMD, mg/mm^2^) were measured using DXA (Piximus 2, Lunar Corporation, Madison, WI). Femur and humerus length (mm) were measured using digital calipers (Marathon Watch Company Ltd., Richmond Hill, Ontario Canada).

### Micro-computed Tomography

μCT was used for nondestructive three-dimensional evaluation of bone volume and cortical and cancellous bone microarchitecture. Femora, humeri, 2^nd^ lumbar vertebrae, and right calvaria were scanned using a Scanco μCT40 scanner (Scanco Medical AG, Basserdorf, Switzerland) at 55 kV_p_ x-ray voltage, 145 μA intensity, and 200 ms integration time. Femora, humeri, and right calvaria were scanned at a voxel size of 12 μm × 12 μm × 12 μm while 2^nd^ lumbar vertebrae were scanned at a voxel size of 16 μm × 16 μm × 16 μm. Filtering parameters sigma and support were set to 0.8 and 1, respectively. The threshold value for evaluation was determined empirically and set at 245 (gray scale, 0-1000). Cortical bone was evaluated in the femur and humerus diaphyses (Fig. 1a,b). Cancellous bone was evaluated in the distal femur metaphysis and epiphysis, in the distal humerus epiphysis, and in the vertebral body (Fig. 1a–c). Calvarial thickness was evaluated in a subsection of the parietal bone (Fig. 1d).

#### Femur

Eighty-two slices (984 μm in length) of cortical bone were assessed in the femur mid-diaphysis. Automated contouring was used to delineate cortical bone from non-bone. Following, all cortical slices were examined visually for evidence of cancellous struts originating from the endocortex (extremely rare at this site) and manually removed when present. Direct cortical bone measurements included total cross-sectional volume (mm^3^), cortical volume (mm^3^), marrow volume (mm^3^) and cortical thickness (μm). Three-dimensional cortical volume measurements were converted to two-dimensional area measurements^53^. Polar moment of inertia (mm^4^) was determined as a surrogate measure of bone strength in torsion. Seventy four slices (888 μm in length) of cancellous bone, 150 slices (1,800 μm in length) proximal to the growth plate, were assessed in the distal femur metaphysis. The entire cancellous bone compartment (64 ± 1 slices, 768 ± 12 μm) was evaluated in the distal femur epiphysis. Direct cancellous bone measurements included bone volume fraction (bone volume/tissue volume; volume of total tissue occupied by cancellous bone, %), connectivity density (number of redundant connections per unit volume, mm^−3^; this index detects defects in cancellous architecture), structure model index (an architectural index defining bone as plate-like or rod-like with values ranging from 0 to 3, respectively), trabecular thickness (mean thickness of individual trabeculae, μm), trabecular number (number of trabecular intercepts per unit length, mm^−1^) and trabecular separation (distance between trabeculae, μm).

### Humerus

Twenty slices (240 μm in length) of cortical bone were evaluated in the humerus diaphysis. The entire cancellous compartment (65 ± 1 slices, 780 ± 12 μm in length) was evaluated in the distal humerus epiphysis. Cortical and cancellous bone measurements were the same as those described for the femur.

### Lumbar vertebra

Analysis of the lumbar vertebra included the entire region of cancellous bone in the vertebral body between the cranial and caudal growth plates (238 ± 2 slices, 3808 ± 32 μm in length). Cancellous bone measurements were the same as those described for the distal femur.

### Calvarium

Calvarial thickness (μm) was measured in the parietal bone in a 1.2 × 2.4 mm rectangle starting 1.8 mm rostral to the lambdoid suture, and midway between the sagittal suture and temporal crest.

### Statistical Analysis

Mean responses were compared between three groups (baseline, ground control, flight) using analysis of variance (ANOVA), while two-group comparisons were made using t-tests. When the assumption of equal variance was violated, Welch’s two-sample t-test was used for two-group comparisons^54^. When the normality assumption was violated, the Wilcoxon-Mann-Whitney test was used for two-group comparisons. The required conditions for valid use of t-tests and ANOVA were assessed using Levene’s test for homogeneity of variance and the Anderson-Darling test of normality. The Benjamini and Hochberg method for maintaining the false discovery rate at 5% was used to adjust for multiple comparisons^55^. Differences were considered significant at p ≤ 0.05. Data are presented as mean ± SE. Data analysis was performed using RStudio version 0.98.1083.

## Additional Information

**How to cite this article**: Keune, J. A. *et al.* Effects of Spaceflight on Bone Microarchitecture in the Axial and Appendicular Skeleton in Growing Ovariectomized Rats. *Sci. Rep.* **5**, 18671; doi: 10.1038/srep18671 (2015).

## Figures and Tables

**Table 1:** Effects of spaceflight on femur length, total femur bone mass and density, cortical bone architecture in the femur diaphysis, and cancellous bone architecture in the distal femur metaphysis and epiphysis.

	Ovary-Intact Reference Rats			Ovariectomized Rats			FDR-adjusted p value
	12-week-old	14-week-old	FDR-adjusted p value	Baseline (12-week-old)	Ground Control (14-week-old)	Flight (14-week-old)	
Length (mm)	28.4 ± 0.2	29.5 ± 0.2	0.01	28.4 ± 0.2	29.4 ± 0.1^a^	29.4 ± 0.1^a^	**<0.001**
Densitometry							
Bone area (mm^2^)	153 ± 2	160 ± 1	0.02	156 ± 2	164 ± 1^a^	161 ± 1	**0.04**
BMC (mg)	198 ± 4	220 ± 5	0.02	182 ± 4	191 ± 3	175 ± 3^b^	**0.01**
BMD (mg/mm^2^)	1.3 ± 0.0	1.4 ± 0.0	0.02	1.2 ± 0.0	1.2 ± 0.0	1.1 ± 0.0^a^,^b^	**<0.001**
microComputed Tomography							
Femur Diaphysis (cortical bone)							
Cross-Sectional Area (mm^2^)	6.1 ± 0.1	6.3 ± 0.1	NS	6.2 ± 0.1	6.3 ± 0.1	6.1 ± 0.1	NS
Cortical Area (mm^2^)	3.6 ± 0.0	3.8 ± 0.0	0.05	3.6 ± 0.1	3.7 ± 0.0	3.6 ± 0.0	0.08
Marrow Area (mm^2^)	2.5 ± 0.0	2.4 ± 0.0	NS	2.6 ± 0.1	2.6 ± 0.0	2.5 ± 0.0	NS
Cortical Thickness (μm)	490 ± 4	512 ± 5	**0.02**	470±3	492 ± 4^a^	478 ± 4	**0.02**
Polar Moment of Inertia (mm^4^)	5.0 ± 0.1	5.3 ± 0.1	NS	5.0 ± 0.2	5.3 ± 0.1	5.0 ± 0.1	NS
Distal Femur Metaphysis (cancellous bone)							
Bone Volume/Tissue Volume (%)	15.3 ± 1.1	18.7 ± 0.8	0.07	8.0 ± 0.4	6.8 ± 0.3	4.9 ± 0.3^a^,^b^	**<0.001**
Connectivity Density (mm^−3^)	92 ± 7	107 ± 5	NS	47 ± 3	37 ± 2	22 ± 2^a^,^b^	**<0.001**
Structure Model Index	2.0 ± 0.1	1.7 ± 0.1	**0.02**	2.3 ± 0.1	2.3 ± 0.0	2.5 ± 0.0^a^,^b^	**<0.001**
Trabecular Number (mm^−1^)	3.5 ± 0.1	3.7 ± 0.2	NS	3.2 ± 0.1	3.1 ± 0.1	2.8 ± 0.1^a^	**0.03**
Trabecular Thickness (μm)	63 ± 1	67 ± 1	**0.02**	59 ± 0	60 ± 1	61 ± 1	NS
Trabecular Spacing (μm)	285 ± 12	268 ± 12	NS	318 ± 5	325 ± 9	357 ± 9^a^	**0.04**
Distal Femur Epiphysis (cancellous bone)							
Bone Volume/Tissue Volume (%)	33.5 ± 0.4	34.0 ± 0.4	NS	31.3 ± 0.6	28.2 ± 0.2^a^	24.1 ± 0.4^a^,^b^	**<0.001**
Connectivity Density (mm^−3^)	81 ± 2	75 ± 2	NS	102 ± 2	88 ± 1^a^	80 ± 1^a^,^b^	**<0.001**
Structure Model Index	0.1 ± 0.1	0.3 ± 0.0	0.07	−0.1 ± 0.1	0.2 ± 0.02^a^	0.5 ± 0.0^a^,^b^	**<0.001**
Trabecular Number (mm^−1^)	4.0 ± 0.1	4.1 ± 0.1	NS	4.0 ± 0.1	3.9 ± 0.03	3.6 ± 0.0^a^,^b^	**<0.001**
Trabecular Thickness (μm)	82 ± 1	83 ± 1	NS	77 ± 1	75 ± 0	70 ± 1^a^,^b^	**<0.001**
Trabecular Spacing (μm)	235 ± 3	235 ± 4	NS	238 ± 3	248 ± 2^a^	265 ± 2^a^,^b^	**<0.001**

Data are mean ± SE.

NS, Not Significant.

^a^Different from Baseline, P ≤ 0.05.

^b^Different from Ground Control, P ≤ 0.05.

**Table 2:** Effects of spaceflight on humerus length, total humerus bone mass and density, cortical bone architecture in the humerus diaphysis, and cancellous bone architecture in the distal humerus epiphysis.

	Ovary-Intact Reference Rats		FDR-adjusted p value	Ovariectomized Rats		FDR-adjusted p value
	12-week-old	14-week-old		Ground Control (14-week-old)	Flight (14-week-old)	
Length (mm)	23.5 ± 0.2	24.3 ± 0.1	**0.01**	24.3 ± 0.1	24.3 ± 0.1	NS
Densitometry						
Bone area (mm^2^)	97 ± 1	102 ± 2	0.07	97 ± 1	97 ± 1	NS
BMC (mg)	93 ± 2	100 ± 2	0.07	89 ± 1	88 ± 1	NS
BMD (mg/mm^2^)	1.0 ± 0.2	1.0 ± 0.1	NS	0.9 ± 0.1	0.9 ± 0.1	NS
microComputed Tomography						
Humerus Diaphysis (cortical bone)						
Cross-Sectional Area (mm^2^)	3.0 ± 0.0	3.0 ± 0.0	NS	3.0 ± 0.0	3.1 ± 0.0	NS
Cortical Area (mm^2^)	2.4 ± 0.0	2.4 ± 0.0	NS	2.4 ± 0.0	2.4 ± 0.0	NS
Marrow Area (mm^2^)	0.7 ± 0.0	0.6 ± 0.0	**0.01**	0.7 ± 0.0	0.7 ± 0.0	**0.01**
Cortical Thickness (μm)	488 ± 5	509 ± 7	0.07	486 ± 4	477 ± 4	NS
Polar Moment of Inertia (mm^4^)	1.4 ± 0.0	1.5 ± 0.0	NS	1.4 ± 0.0	1.5 ± 0.0	NS
Distal Humerus Epiphysis (cancellous bone)						
Bone Volume/Tissue Volume (%)	37.9 ± 0.7	38.2 ± 0.4	NS	32.6 ± 0.5	32.6 ± 0.3	NS
Connectivity Density (mm^−3^)	74 ± 2	69 ± 2	NS	95 ± 2	96 ± 2	NS
Structure Model Index	−0.4 ± 0.1	−0.5 ± 0.0	NS	0.0 ± 0.0	0.0 ± 0.0	NS
Trabecular Number (mm^−1^)	4.0 ± 0.1	3.9 ± 0.1	NS	3.9 ± 0.0	3.8 ± 0.1	NS
Trabecular Thickness (μm)	91 ± 2	92 ± 1	NS	80 ± 1	82 ± 0	NS
Trabecular Spacing (μm)	227 ± 5	235 ± 6	NS	240 ± 3	249 ± 4	NS

Data are mean ± SE.

NS, Not Significant.

**Table 3:** Effects of spaceflight on cancellous bone architecture in the second lumbar vertebra and cortical thickness of the parietal bone in calvarium.

	Ovary-Intact Reference Rats			Ovariectomized Rats		
	12-week-old	14-week-old	FDR-adjusted p value	Ground Control (14-week-old)	Flight (14-week-old)	FDR-adjusted p value
2nd Lumbar Vertebra (cancellous bone)						
Bone Volume/Tissue Volume (%)	30.3 ± 0.6	32.1 ± 0.7	NS	20.9 ± 0.4	16.9 ± 0.8	**<0.001**
Connectivity Density (mm^−3^)	87 ± 2	76 ± 2	**0.02**	68 ± 2	65 ± 2	NS
Structure Model Index	0.2 ± 0.0	−0.2 ± 0.1	**0.02**	0.9±0.0	1.3 ± 0.1	**<0.001**
Trabecular Number (mm^−1^)	4.0 ± 0.1	3.9 ± 0.1	NS	3.3 ± 0.1	3.1 ± 0.1	**0.01**
Trabecular Thickness (μm)	76 ± 1	79 ± 1	NS	68 ± 1	62 ± 1	**0.00**
Trabecular Spacing (μm)	231 ± 4	239 ± 5	NS	294 ± 6	325 ± 6	**0.00**
Calvarium						
Parietal Thickness (μm)				346 ± 8	342 ± 6	NS

Data are mean ± SE.

NS, Not Significant.

## References

[b1] Turner C. H. Three rules for bone adaptation to mechanical stimuli. Bone 23, 399–407 (1998). 9823445 10.1016/s8756-3282(98)00118-5

[b2] Meakin L. B., Price J. S. & Lanyon L. E. The Contribution of Experimental *in vivo* Models to Understanding the Mechanisms of Adaptation to Mechanical Loading in Bone. Front Endocrinol (Lausanne) 5, 154 (2014). 25324829 10.3389/fendo.2014.00154 PMC4181237

[b3] Orwoll E. S. *et al.* Skeletal health in long-duration astronauts: nature, assessment, and management recommendations from the NASA Bone Summit. J Bone Miner Res 28, 1243–1255 (2013). 23553962 10.1002/jbmr.1948

[b4] Sibonga J. D. Spaceflight-induced bone loss: is there an osteoporosis risk? Curr Osteoporos Rep 11, 92–98 (2013). 23564190 10.1007/s11914-013-0136-5

[b5] Turner R. T. Invited review: what do we know about the effects of spaceflight on bone? J Appl Physiol (1985) 89, 840–847 (2000). 10926671 10.1152/jappl.2000.89.2.840

[b6] Rittweger J. *et al.* Muscle atrophy and bone loss after 90 days’ bed rest and the effects of flywheel resistive exercise and pamidronate: results from the LTBR study. Bone 36, 1019–1029 (2005). 15811637 10.1016/j.bone.2004.11.014

[b7] Morey E., Turner R. & Baylink D. The effect of spaceflight on selected bone parameters. (Neuka Press, 1979).

[b8] Zhang B., Cory E., Bhattacharya R., Sah R. & Hargens A. R. Fifteen days of microgravity causes growth in calvaria of mice. Bone 56, 290–295 (2013). 23791778 10.1016/j.bone.2013.06.009 PMC4110898

[b9] Ronca A. E. *et al.* Effects of sex and gender on adaptations to space: reproductive health. J Womens Health (Larchmt) 23, 967–974 (2014). 25401943 10.1089/jwh.2014.4915 PMC4235591

[b10] Tou J. C., Grindeland R. E. & Wade C. E. Effects of diet and exposure to hindlimb suspension on estrous cycling in Sprague-Dawley rats. Am J Physiol Endocrinol Metab 286, E425–433 (2004). 14625203 10.1152/ajpendo.00287.2003

[b11] Westerlind K. C. *et al.* Estrogen regulates the rate of bone turnover but bone balance in ovariectomized rats is modulated by prevailing mechanical strain. Proc Natl Acad Sci USA 94, 4199–4204 (1997). 9108129 10.1073/pnas.94.8.4199 PMC20601

[b12] Lin B. Y. *et al.* Mechanical loading modifies ovariectomy-induced cancellous bone loss. Bone Miner 25, 199–210 (1994). 8086858 10.1016/s0169-6009(08)80239-5

[b13] Turner R. T., Hannon K. S., Demers L. M., Buchanan J. & Bell N. H. Differential effects of gonadal function on bone histomorphometry in male and female rats. J Bone Miner Res 4, 557–563 (1989). 2816504 10.1002/jbmr.5650040415

[b14] Strollo F. *et al.* The pituitary-testicular axis in microgravity: analogies with the aging male syndrome. J Endocrinol Invest 28, 78–83 (2005). 16760631

[b15] Smith S. M., Heer M., Wang Z., Huntoon C. L. & Zwart S. R. Long-duration space flight and bed rest effects on testosterone and other steroids. J Clin Endocrinol Metab 97, 270–278 (2012). 22049169 10.1210/jc.2011-2233 PMC3251930

[b16] Wimalawansa S. M. & Wimalawansa S. J. Simulated weightlessness-induced attenuation of testosterone production may be responsible for bone loss. Endocrine 10, 253–260 (1999). 10484289 10.1007/BF02738624

[b17] Turner R. T., Vandersteenhoven J. J. & Bell N. H. The effects of ovariectomy and 17 beta-estradiol on cortical bone histomorphometry in growing rats. J Bone Miner Res 2, 115–122 (1987). 3455160 10.1002/jbmr.5650020206

[b18] Wronski T. J., Cintron M. & Dann L. M. Temporal relationship between bone loss and increased bone turnover in ovariectomized rats. Calcif Tissue Int 43, 179–183 (1988). 3141020 10.1007/BF02571317

[b19] Wronski T. J., Lowry P. L., Walsh C. C. & Ignaszewski L. A. Skeletal alterations in ovariectomized rats. Calcif Tissue Int 37, 324–328 (1985). 3926284 10.1007/BF02554882

[b20] Sibonga J. D., Dobnig H., Harden R. M. & Turner R. T. Effect of the high-affinity estrogen receptor ligand ICI 182,780 on the rat tibia. Endocrinology 139, 3736–3742 (1998). 9724025 10.1210/endo.139.9.6172

[b21] Wimalawansa S. M. *et al.* Reversal of weightlessness-induced musculoskeletal losses with androgens: quantification by MRI. J Appl Physiol (1985) 86, 1841–1846 (1999). 10368347 10.1152/jappl.1999.86.6.1841

[b22] Bullard T. R. & Lindsay H. A. Age as a determinant of the effect of testosterone upon disuse osteoporosis of paralyzed limbs of rats. Can J Physiol Pharmacol 46, 689–691 (1968). 5668216 10.1139/y68-103

[b23] Yarrow J. F. *et al.* A rehabilitation exercise program induces severe bone mineral deficits in estrogen-deficient rats after extended disuse. Menopause 19, 1267–1276 (2012). 22713863 10.1097/gme.0b013e318255657f PMC8422355

[b24] Cavolina J. M. *et al.* The effects of orbital spaceflight on bone histomorphometry and messenger ribonucleic acid levels for bone matrix proteins and skeletal signaling peptides in ovariectomized growing rats. Endocrinology 138, 1567–1576 (1997). 9075717 10.1210/endo.138.4.5040

[b25] Turner R. T., Riggs B. L. & Spelsberg T. C. Skeletal effects of estrogen. Endocr Rev 15, 275–300 (1994). 8076582 10.1210/edrv-15-3-275

[b26] Rubin C. T., Gross T. S., McLeod K. J. & Bain S. D. Morphologic stages in lamellar bone formation stimulated by a potent mechanical stimulus. J Bone Miner Res 10, 488–495 (1995). 7785471 10.1002/jbmr.5650100321

[b27] Bagi C. M. & Miller S. C. Comparison of osteopenic changes in cancellous bone induced by ovariectomy and/or immobilization in adult rats. Anat Rec 239, 243–254 (1994). 7943756 10.1002/ar.1092390303

[b28] Backup P. *et al.* Spaceflight results in reduced mRNA levels for tissue-specific proteins in the musculoskeletal system. Am J Physiol 266, E567–573 (1994). 8178977 10.1152/ajpendo.1994.266.4.E567

[b29] Bateman T. A. *et al.* Histomorphometric, physical, and mechanical effects of spaceflight and insulin-like growth factor-I on rat long bones. Bone 23, 527–535 (1998). 9855461 10.1016/s8756-3282(98)00135-5

[b30] Morey E. R. & Baylink D. J. Inhibition of bone formation during space flight. Science 201, 1138–1141 (1978). 150643 10.1126/science.150643

[b31] Westerlind K. C. & Turner R. T. The skeletal effects of spaceflight in growing rats: tissue-specific alterations in mRNA levels for TGF-beta. J Bone Miner Res 10, 843–848 (1995). 7572306 10.1002/jbmr.5650100603

[b32] Wronski T. J. & Morey E. R. Effect of spaceflight on periosteal bone formation in rats. Am J Physiol 244, R305–309 (1983). 6402940 10.1152/ajpregu.1983.244.3.R305

[b33] Wronski T. J., Morey-Holton E. R., Doty S. B., Maese A. C. & Walsh C. C. Histomorphometric analysis of rat skeleton following spaceflight. Am J Physiol 252, R252–255 (1987). 3812763 10.1152/ajpregu.1987.252.2.R252

[b34] Turner R. T., Evans G. L. & Wakley G. K. Spaceflight results in depressed cancellous bone formation in rat humeri. Aviat Space Environ Med 66, 770–774 (1995). 7487811

[b35] Zerath E. *et al.* Effects of spaceflight and recovery on rat humeri and vertebrae: histological and cell culture studies. J Appl Physiol (1985) 81, 164–171 (1996). 8828659 10.1152/jappl.1996.81.1.164

[b36] Patterson-Buckendahl P. *et al.* Fragility and composition of growing rat bone after one week in spaceflight. Am J Physiol 252, R240–246 (1987). 3812761 10.1152/ajpregu.1987.252.2.R240

[b37] Shaw S. R., Vailas A. C., Grindeland R. E. & Zernicke R. F. Effects of a 1-wk spaceflight on morphological and mechanical properties of growing bone. Am J Physiol 254, R78–83 (1988). 3337273 10.1152/ajpregu.1988.254.1.R78

[b38] Vailas A. C. *et al.* Effects of spaceflight on rat humerus geometry, biomechanics, and biochemistry. FASEB J 4, 47–54 (1990). 2295378 10.1096/fasebj.4.1.2295378

[b39] Lafage-Proust M. H. *et al.* Space-related bone mineral redistribution and lack of bone mass recovery after reambulation in young rats. Am J Physiol 274, R324–334 (1998). 9486288 10.1152/ajpregu.1998.274.2.R324

[b40] Vico L., Bourrin S., Genty C., Palle S. & Alexandre C. Histomorphometric analyses of cancellous bone from COSMOS 2044 rats. J Appl Physiol (1985) 75, 2203–2208 (1993). 8307880 10.1152/jappl.1993.75.5.2203

[b41] Foldes I., Kern M., Szilagyi T. & Oganov V. S. Histology and histochemistry of intervertebral discs of rats participated in spaceflight. Acta Biol Hung 47, 145–156 (1996). 9123987

[b42] Wronski T. J., Dann L. M. & Horner S. L. Time course of vertebral osteopenia in ovariectomized rats. Bone 10, 295–301 (1989). 2803866 10.1016/8756-3282(89)90067-7

[b43] Davis B. A. *et al.* Collagenase and tissue plasminogen activator production in developing rat calvariae: normal progression despite fetal exposure to microgravity. Calcif Tissue Int 63, 416–422 (1998). 9799827 10.1007/s002239900550

[b44] Hatton D. C. *et al.* Calcium metabolism and cardiovascular function after spaceflight. J Appl Physiol (1985) 92, 3–12 (2002). 11744636 10.1152/jappl.2002.92.1.3

[b45] Simmons D. J., Grynpas M. D. & Rosenberg G. D. Maturation of bone and dentin matrices in rats flown on the Soviet biosatellite Cosmos 1887. FASEB J 4, 29–33 (1990). 2153084 10.1096/fasebj.4.1.2153084

[b46] Zhang M. & Turner R. T. The effects of spaceflight on mRNA levels for cytokines in proximal tibia of ovariectomized rats. Aviat Space Environ Med 69, 626–629 (1998). 9681367

[b47] Hefferan T. E. *et al.* Effect of gender on bone turnover in adult rats during simulated weightlessness. J Appl Physiol (1985) 95, 1775–1780 (2003). 12882994 10.1152/japplphysiol.00455.2002

[b48] Morey E. R., Sabelman E. E., Turner R. T. & Baylink D. J. A new rat model simulating some aspects of space flight. Physiologist 22, S23–24 (1979). 545372

[b49] Turner R. T., Evans G. L., Cavolina J. M., Halloran B. & Morey-Holton E. Programmed administration of parathyroid hormone increases bone formation and reduces bone loss in hindlimb-unloaded ovariectomized rats. Endocrinology 139, 4086–4091 (1998). 9751486 10.1210/endo.139.10.6227

[b50] Tavella S. *et al.* Bone turnover in wild type and pleiotrophin-transgenic mice housed for three months in the International Space Station (ISS). PLoS One 7, e33179 (2012). 22438896 10.1371/journal.pone.0033179 PMC3305296

[b51] Blaber E. A. *et al.* Microgravity induces pelvic bone loss through osteoclastic activity, osteocytic osteolysis, and osteoblastic cell cycle inhibition by CDKN1a/p21. PLoS One 8, e61372 (2013). 23637819 10.1371/journal.pone.0061372 PMC3630201

[b52] Turner R. T. & Iwaniec U. T. Low dose parathyroid hormone maintains normal bone formation in adult male rats during rapid weight loss. Bone 48, 726–732 (2011). 21215827 10.1016/j.bone.2010.12.034 PMC3062670

[b53] Bouxsein M. L. *et al.* Guidelines for assessment of bone microstructure in rodents using micro-computed tomography. J Bone Miner Res 25, 1468–1486 (2010). 20533309 10.1002/jbmr.141

[b54] Welch B. On the comparison of several mean values: an alternative approach. Biometrika 38, 330–336 (1951).

[b55] Benjamini Y. & Hochberg Y. Controlling the false discovery rate: a practical and powerful approach to multiple testing. J Royal Statistical Society Series B 57, 289–900 (1995).

